# Supplementary material for: CCL4 Functions as a Biomarker of Type 2 Airway Inflammation
Source: Biomedicines. 2022 Jul 23;10(8):1779. doi: 10.3390/biomedicines10081779 (PMC9330411; doi:10.3390/biomedicines10081779)
Supplement: Supplementary file 1 [file biomedicines-10-01779-s001.zip › biomedicines-1812184-supplementary.pdf]

Table S1. Amplification primers (5′–3′)

gene	forward	reverse
TSLP	CCG CCT ATG AGC AGC CAC	CCT CAG TAG CAT TTA TCT CAG
IL-25	GAG ATA TGA GTT GGA CAG AGA CTT GAA	CCA TGT GGG AGC CTG TCT GTA
IL-33	GAA GAA CAC AGC AAG CAA AGC	TAC CAA AGG CAA AGC ACT CC
CCL4	CTC CTC ATG CTA GTA GCT GCC TTC	GGT GTA AGA AAA GCA GCA GGC GGT
CCL5	GTT GCA CCA AGC TAT GCA GGT	GCA GAA GCG TTT GGC AAT GT
CCL11	CCA CAC TGA AGG TCT CCG CA	TCT CTA GTC GCT GAA GGG GT
CCL26	ACT CCG AAA CAA TTG TGA CTC AGC TG	GTA ACT CTG GGA GGA AAC ACC CTC TCC
TSLPR	ATA CTC CAG GAC CAA CCT GAC	ACA CGT CAC CGT CAC TGC ATC
TLR3	GGA TAG CTC TCC TTC ACC ATT C	GAC CCT CCA ACA TGT CAT CAT
CCR5	CCC AGT GCA CAC AAG TGT AGG TAT C	GCT CAA GGC AGC TTA TTT CCA A
ICAM-1	CTG CAG ACA GTG ACC ATC	GTC CAG TTT CCC GGA CAA
GAPDH	TTC ACC ACC ATG GAG AAG GC	AGG AGG CAT TGC TGA TGA TCT

Table S2. Characteristics of patients treated with dupilumab (n=19)

Age (years)	55.2 ± 14.9	FENO (ppb)	70.5 ± 44.7
Gender (M/F)	11/8	Lund-Mackay scale	14.5 ± 5.9
Body Mass Index (kg/m^2)	22.4 ± 3.3	Polyp score	4.9 ± 2.3
NSAID intolerance	7	JESREC score	15.9 ± 1.0
Smoking status (never/ex)	14/5	FEV_1 (%predicted)	83.2 ± 19.8
ESS history (Y/N)	17/2	FEF_{25-75} (%predicted)	57.5 ± 38.0
Total IgE (IU/mL)	741 ± 797	FVC (%predicted)	96.3 ± 10.8
Positive RAST (none/single/multi)	5/3/11	Asthma exacerbation (per year)*	1.2 ± 1.8
Eosinophils (/µL) [peak value]	446 ± 369 [895 ± 528]		
Treatment			
Inhaled corticosteroids (µg)**	1047 ± 176	Anti-histamine	11
Long-acting β_2-adrenergic agonist	19	Inhaled nasal corticosteroids	14
Long-acting muscarinic antagonist	8	Oral corticosteroids	1
Leukotriene receptor antagonist	12	Low-dose macrolide	2

ESS: endoscopic sinus surgery; FENO = fractionated exhaled nitrogen oxide; FEF_{25-75}: forced expiratory flow between 25% and 75% of vital capacity; FEV_1: forced expiratory volume in 1 second; FVC: forced vital capacity; JESREC: Japanese Epidemiological Survey of Refractory Eosinophilic Chronic Rhinosinusitis. *A clinically significant exacerbation defined as any worsening of asthma considered by the treating physician to require systemic corticosteroids. ** Equivalent doses of fluticasone propionate. Values are number of subjects and mean ± standard deviation.
